# Lifeboat Saturday

**Published:** 1892-08-06

**Authors:** Marion Macara

**Affiliations:** St. Anne's-on-Sea


### EDITOR'S LETTER BOX.

[Our correspondents are reminded that prolixity of statement is a great bar to publication, and that brevity of style and conciseness of statement greatly facilitate early insertion.]

### "LIFEBOAT SATURDAY."

Sir,—The earnest appeal to the women of England to help in the lifeboat cause which has been made by Mr. E. G. McConnel, of Manchester, will, I feel sure, meet with even a more hearty response than his last year's one entitled, "Women and Children First." The graphic way in which he describes his own experience of seeing a lifeboat put out to sea in a whole gale against wind and tide, must have touched many a woman's heart. I, too, have seen a lifeboat launched in a fearful storm, and have waited and watched with the wives and mothers of the crew, all through the long hours of a wild December night, for the return of the brave men who went out to save, but, alas, returned no more. Such are the sacrifices which the humble homes of our fisher-folk are called upon from time to time to make. Surely the wives and daughters of England will not be behind in doing their share towards the maintenance of this noble volunteer force. I am glad to see that Lady Roscoe is taking a lead in this matter. If a Central Committee of ladies could be organised in the North of England and another in the South, with sub-committees in each town to collect small sums from the many in aid of the "Lifeboat Saturday" Fund, I think it would be a great assistance in commencing the movement where it has not been taken up, and also in rendering it a permanent success.—Yours truly,

Marion Macara.

